# Production of non-activated biochar based on *Biden pilosa* and its application in removing methylene blue from aqueous solutions

**DOI:** 10.1016/j.heliyon.2023.e15766

**Published:** 2023-04-25

**Authors:** Supin Sangsuk, Pinanong Napanya, Siwabhorn Tasen, Phannida Baiya, Chatchai Buathong, Khemissara Keeratisoontornwat, Sirisak Suebsiri

**Affiliations:** a School of Agricultural Resources, Chulalongkorn University, Phayathai Rd., Pathumwan, Bangkok, 10330, Thailand; b Fiber Resource Energy Cooporation Ltd., Klangdong, Pakchong District, Nakorn Rachasima, 30320, Thailand

**Keywords:** Charcoal kiln, Pyrolysis, *Biden pilosa*, Adsorption, Methylene blue

## Abstract

*Biden pilosa* (BP) is a type of weed commonly found in Thailand that needs to be removed from agricultural areas for protecting main crops. This research proposed a method to reduce BP by using BP as a feedstock for biochar production. Non-activated BP biochar from fresh BP was produced in pilot scale using a drum kiln with a heat-transferring duct at a pyrolysis temperature of 550 °C at a slow heating rate. The physical properties of the non-activated BP biochar were investigated using scanning electron microscopy, Fourier transform infrared (FTIR) spectroscopy, X-ray diffraction, and a surface area analyzer. A batch experiment was used to study the adsorption behavior of methylene blue (MB) on BP biochar. The microstructure study of the BP biochar indicated that it has a cell structure similar to that of BP, which shows the non-destructive nature of the proposed technique for BP production. Six dominant peaks at 3283, 2915, 1559, 1403, 1116, and 863/839 cm^⁻1^ were observed in the FTIR spectrum. The BP biochar exhibited a surface area of 5.21 m^2^/g and a pore size of 8 nm. The adsorption of MB on the BP biochar followed the Langmuir adsorption isotherm and pseudo-second-order kinetics. The Langmuir-based maximum adsorption capacity of MB on the BP biochar was 200 mg/g at 303 K.

## Highlights

• Low-cost BP biochar production on a pilot scale using a cheap device and feedstock.
• Save energy for feedstock transportation when biochar was produced in a local area.
• Non-activated BP biochar exhibited a high MB adsorption capacity.

## Graphical abstract

**Figure:**
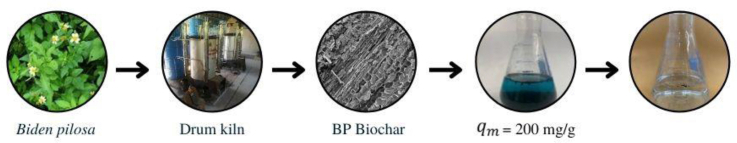

## 1 Introduction

Biden pilosa (BP) originated from South Africa and spread to all tropical and subtropical regions of the world, especially in Thailand. This can be attributed to its hardiness, adaptability to most environments, and significant reproductive capability [1]. BP is typically regarded as a weed because it rapidly spreads even on disturbed ground, harming several other vegetations. Thus, to preserve valuable plants, Thai farmers dispose of it by mowing or applying herbicides. BP is not suitable for use as animal feed because its seed is as sharp as a thorn, and cutting BP would leave their seeds in the field to germinate under suitable conditions. Therefore, using BP as a feedstock for biochar production is a potential method for reducing the amount of BP. Consequently, farmers reduce their expense for herbicides, which is good for the environment. Considering its suitability with external physical characteristics, BP biochar is more suitable for use as an adsorbent because it is fragile and easily reduced in size.

Water contamination is a significant environmental problem that requires great attention. Numerous techniques have been investigated to remove contaminants from water. Adsorption technology can remove pollutants from a solvent phase to the solid phase [2]. For both organic and inorganic pollutants in water, carbonaceous materials have long been used as adsorbents for the adsorption process. Both activated carbon and biochar are carbonaceous materials produced using pyrolysis. Activated carbon is produced from both wood and coal [3], whereas biochar is produced from biomass, such as agricultural waste and forestry residues, including animal manure [4].

Organic micropollutants can be successfully removed using activated carbon, but producing activated carbon is expensive and energy-intensive. Biochar based adsorption can save money [5]. Biochar is generated by the pyrolysis of biomass at a low temperature (approximately 700 °C) in an oxygen-limited environment. Due to variations in the pyrolysis conditions, i.e., the raw materials and technological procedures used to produce biochar, the final product has different physicochemical properties [6]. An advantage of biochar as an environmental technology is its capacity to immobilize pollutants [7]. Several contaminants, including dyes, heavy metals, pesticides, and antibiotic pollutants, can be effectively and efficiently adsorbed on biochar because of its surface area and pore structure, functional groups, and hydrophobic properties [8].

Dyes prevent light from penetrating streams and rivers, which inhibits photosynthesis and reduces the concentration of dissolved oxygen [9]. Dyes pose a major health risk to people, especially those who are hypersensitive; allergic; or who have asthma, renal, liver, or brain disorders [10]. Methylene blue (MB) is a group of cationic dyes used in several coloring and dyeing processes, in biological and pharmaceutical processes, and as chemical indicators [11]. MB is detrimental both to the environment and people.

Studies on MB reduction in water using activated carbon and biochar based on biomass wastes are interesting because of their adsorption capacity and the different biomass obtained from different local areas. Activated carbon for MB adsorption can be obtained from various agricultural wastes and biological raw materials such as longan seeds [12] and *Dipterocarpus alatus* fruits [13]. Biochar for MB adsorption can be obtained from several sources, including seaweed [8], date palm fronds [14], areca leaf [15], and sugarcane bagasse [16].

Although biochar is useful and interesting to study, different production methods result in different biochar properties, which are affected by biomass types and pyrolysis temperature. The biomass type in local areas is a choice of raw materials for biochar production. Several studies also showed the effect of pyrolysis temperature on biochar properties. For example, Suhaimi research [17] produced bamboo biochar at various pyrolysis temperatures (400°C-800 °C). Results showed that biochars pyrolyzed at 500 °C had the highest adsorptive performance for MB from an aqueous solution. The biochar preparation technique is one of the key conditions affecting the properties of biochar.

According to Li summarization [18], there were five possible adsorption mechanisms: π-π interactions (π-π electron-donor-acceptor), hydrogen bonds, electrostatic interactions, hydrophilic interactions, and pore filling. For π-π interactions, the functional groups on biochar act as electron donors, such as C=C or –OH.

In hydrogen bonds, the important functional groups are phenolic hydroxyl, carbonyl, and anhydride to form hydrogen bonds with dye contaminants. Electrostatic interactions involve the adsorption of ionic and ionizable contaminants, surface charges of an adsorbent, and pH solution. Generally, biochar has a negative charge, so its adsorption of cationic dyes follows an electrostatic interaction mechanism via a functional group on its surface to form a complex with cationic dye [19].

Furthermore, the pH of contaminant solutions affects the affinity between biochar (adsorbents) and dyes (adsorbates). Sackery research [20] showed that the adsorption capacity of bamboo biochar and rice straw biochar on basic red dyes increased steadily in the pH range of <7 and sharply at pH 9–11.

Hydrophobic interactions in biochar were observed at low pyrolysis temperatures, forming an aliphatic domain, which is a part of hydrophobic organic compounds.

Finally, pore filling is a possible adsorption mechanism. It is related to the surface properties of biochar, whose capacity is directly proportional to its surface area [18].

This study aimed to eliminate BP from available agricultural areas in Thailand and to produce BP biochar. Fresh BP was pyrolyzed in a high-energy conversion efficiency charcoal drum kiln with a heat-transferring duct. This is the first biochar production from fresh BP on a pilot scale. The physical properties and adsorption abilities of non-activated BP biochar were characterized. The kinetic and equilibrium adsorption of MB on BP biochar in aqueous solutions were evaluated under batch conditions.

## 2 Experimental

### 2.1 Biochar production

BP was used as the raw material in this experiment. Fresh BP was obtained from the field at the Sufficiency Agricultural Practice Center, Chulalongkorn University, Nan Province, Thailand.

Fresh BP was cut using grass shears and collected, excluding the roots. It was not washed to avoid excess water, and it was used without reducing the size before weighing and loading in a drum kiln with a heat-transferring duct. The kiln is a type of high-energy conversion efficiency kiln that was designed for charcoal and biochar production [21]. The BP moisture content was estimated following method for the determination of moisture content [22]. The cell structure of fresh BP was characterized using an optical microscope (Optika, B-190TBPL).

After loading 22 kg of fresh BP into the kiln, its steel lid was closed and fastened with a metal ring. Firewood was used as a heat source, and ignition from the firing place was done.

During BP biochar production, the temperature changes within the kiln were recorded. No smoke came out from the chimneys; the air intake at the chimneys and fire area was blocked to stop the heating process and cool down the kiln.

Unlike electric kilns, the production temperature was not set before starting the process. The peak pyrolysis temperature resulted from the carbonization process within the drum, which depended on feedstock types.

**Fig. 1:**
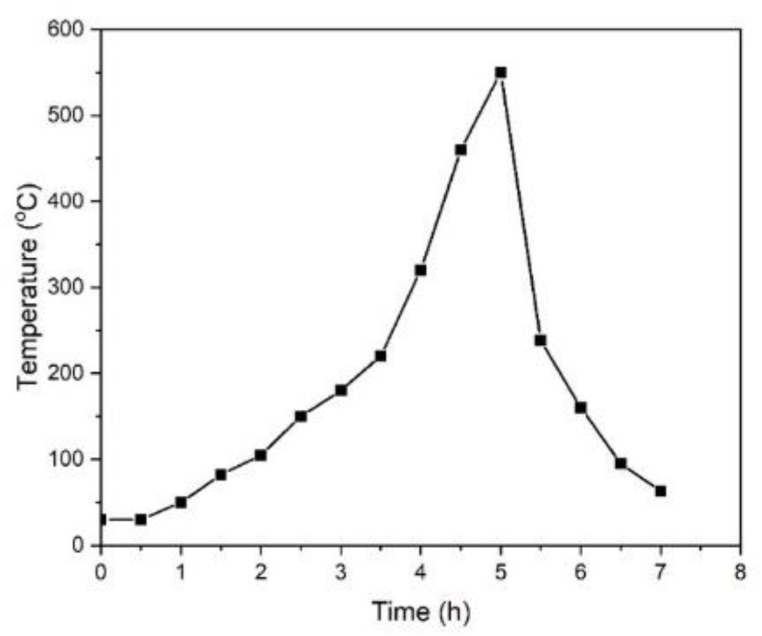
Temperature gradient during the manufacturing process of the *Biden pilosa* biochar in the drum kiln with a heat-transferring duct.

The temperature changes during biochar production were recorded over the entire process. In the dehydration step, the temperature reached 190 °C after 3 h with a 60 °C/h (1 °C/min) heating rate. The heat rate then increased to approximately 180 °C/h (3 °C/min), and the temperature reached 550 °C after another 2 h. During the cooling down process, the temperature inside the kiln dropped below 50 °C after 2 h (Fig. 1). The moisture content of BP was 88.14% ± 1.49%. Therefore, 2.64 kg of dried BP were obtained from the 22 kg of fresh BP. BP biochar (0.81 kg) was obtained, i.e., a 30% BP biochar yield was achieved.

The non-activated biochar was crushed and sieved through a 250-μm sieve. It was used without washing before adsorption studies.

### 2.2 Biochar characterization

The surface area, pore volume, and pore size of the BP biochar were measured by a Brunauer, Emmett, and Teller (BET) surface analyzer (BET, Micromeritics Flex Version 6.01) at 77 K. The BET equation was applied at a relative pressure (P/P_o_) of approximately 0.3 to enable the evaluation of the BET surface area based on the N_2_ adsorption isotherm.

Scanning electron microscopy (SEM, Hitachi SU3500) was used to examine the microstructure of BP biochars. Before characterization, the samples were coated with gold/palladium (80/20%).

The surface functional groups of the BP biochar were detected by Fourier transform infrared (FTIR) spectroscopy (Thermo Fisher Scientific, Nicolet iZ10). The FTIR spectrometer was equipped with an attenuated total reflection sampling module. The conditions of the spectrometer were in the range of 400–4000 cm^−1^ with 64 scans and 4 cm^−1^ resolution.

Phase analysis of BP biochar was conducted by X-ray diffraction (XRD) using a Bruker model D8 Advance device.

### 2.3 Adsorption studies

MB was obtained from RANKEM (dye content 95%, reagent grade), India, and was used without further purification. Here, MB solutions (50 ml) were placed in 125-ml Erlenmeyer flasks at different starting concentrations (50–500 mg/l), and adsorption isotherms were obtained. Powdered BP biochar (0.1 g) was weighed and mixed with the MB solution.

For accuracy, the number of experiments for each starting MB concentration was repeated three times, and the average was taken.

The mixtures were shaken using an orbital shaker (IKA, KS4000i control) at a constant speed of 120 rpm for the required contact time. A centrifuge (Hettich, Universal 320R) was used for 10 min at 9000 rpm to separate the BP biochar after collecting the aqueous samples at the current time intervals. Spectrophotometric analysis was conducted at 650 nm to calculate the residual concentration of the MB solution (Biochrom, EZ Read 400).

Equation (1) was used to calculate the adsorbed amount at time, t, (q_t_, mg/g), and Equation (2) was used to obtain the adsorbed amount at equilibrium, q_e_ (mg/g).

(1) $$q_{t}=\frac{\left(C_{0}-C_{t}\right)V}{W}$$

(2) $$q_{e}=\frac{\left(C_{0}-C_{e}\right)V}{W}$$

where C_0_ and C_t_ (mg/L) are the MB initial concentration and liquid-phase concentration at time, t, respectively, C_e_ (mg/L) is the equilibrium liquid-phase concentration of MB, W is the mass of the dry adsorbent (g), and V is the volume of the solution (L).

To investigate the effect of pH of 2–11, the pH of MB at 200 mg/l was adjusted with 0.1 M hydrochloric acid solution (RCI Labscan, min. 37%, AR grade) and 0.1 M sodium hydroxide solution (J.T. Baker, 98% purity, reagent grade). A calibrated pH meter (Eutech Instruments pH510) was used for pH determination.

### 2.4 Adsorption analysis

#### 2.4.1 Adsorption isotherm model

Langmuir isotherm and the Freundlich isotherm are two models used to explain the adsorption relationship between adsorbates and adsorbents. The Langmuir isotherm is used for monolayer adsorption of adsorbate on an adsorbent surface. The Freundlich isotherm is used for multilayer adsorption and non-ideal adsorption on heterogeneous surfaces [23,24].

Equation (3) is the linear form of the Langmuir isotherm equation.

(3) $$\frac{C_{e}}{q_{e}}=\frac{1}{q_{m}K_{L}}+\left(\frac{1}{q_{m}}\right)C_{e}$$

where q_m_ (mg/g) is the maximum adsorption capacity of the monolayer, and K_L_ is the Langmuir equilibrium constant.

The logarithmic form of the Freundlich equation can be expressed by Eq. (4).

(4) $$\log\;q_{e}=\log\;K_{F}+\left(\frac{1}{n}\right)\log\;C_{e}$$

where K_F_ ((mg/g) (L/mg))^1/n^ and n are the Freundlich constants.

#### 2.4.2 Adsorption kinetics model

The pseudo-first order equation provided by Lagergren and Svenska [25] serves as the basis for calculating the adsorption rate constant using Eq (5).

(5) $$\ln\left(q_{e}-q_{t}\right)=\ln\;q_{e}-k_{1}t$$

where k_1_ (h^−1^) is the pseudo-first order rate, and t (h) is time.

The pseudo-second-order Ho equation [26] in its linear form is given in Eq (6).

(6) $$\frac{t}{q_{t}}=\frac{1}{k_{2}q_{e}^{2}}+\left(\frac{1}{q_{e}}\right)t$$

where k_2_ (g/mg h) is the rate constant of the pseudo-second-order model.

The root mean square error (RMSE) is estimated by Eq. (7), and confirms the applicability of both kinetic models [2].

(7) $$RMSE=\sqrt{\frac{1}{n-1}\sum_{i=0}^{n}{\left(q_{e,\exp}-q_{e,cal}\right)}^{2}}$$

where N is the total number of data points.

#### 2.4.3 Thermodynamic study

The thermodynamic parameters for the adsorption process were determined based on Eqs. (8), (9), (10) [27].

(8) $$\Delta G^{0}=-RT\;In\;K_{D}$$

(9) $$\Delta G^{0}=\Delta H^{0}-T\Delta S^{0}$$

(10) $$InK_{D}=\frac{-\Delta G^{0}}{RT}=\frac{\Delta S^{0}}{R}-\frac{{\Delta H}^{0}}{R}\frac{1}{T}$$

where T is the absolute temperature (K), R is the universal gas constant (8.314 × 10^−3^ kJ/mol K), G^0^ is the change in the free energy (kJ/mol), H^0^ is the change in the enthalpy (kJ/mol), S^0^ is the change in the entropy (J/mol K), and K_D_ = (q_e_/C_e_) is the single point or linear sorption coefficient.

## 3 Result and discussion

### 3.1 Biochar characteristics

The surface area, total volume and average size of the pores of BP biochar were calculated via the BET approach to be 5.21 m^2^/g, 0.018 cm^3^/g, and 8 nm, respectively. Sun and colleagues [28] reported that the distribution of macropores, mesopores, and micropores significantly affects the adsorption behavior and use of activated carbon. Here, the pore size of the produced BP biochar was within the mesopore size range (8 nm). The mesopore structure of BP biochar promoted the dye adsorption [29]. Large pore volumes and mesopores are crucial for achieving high MB removal [30].

Elnour and colleagues [31] reported that the surface area of the date palm biochar produced at 400 °C were 5.5 m^2^/g. Sackery and colleagues [20] reported that the surface area, of rice straw non-activated biochar prepared at 500 °C were 5.21 m^2^/g. When bamboo biochar was prepared under the same conditions of the rice stalk biochar, it exhibited a surface area of 1.99 m^2^/g.

Xiao and colleagues [32] reported the pyrolysis of halophytes at 500 °C to generate biochar without activation. The specific surface area of a biochar made from *Suaeda altissima* was only 3.5 m^2^/g, whereas that of a biochar made from *Phragmites australis* was 344.02 m^2^/g. Therefore, apart from the pyrolysis temperature, different types of plants generate biochars with different surface areas.

**Fig. 2:**
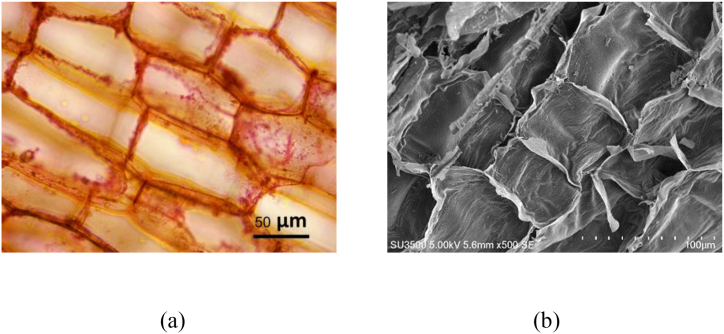
Micrographs of (a) *Biden pilosa* (BP, × 400), (b) BP biochar ( × 500).

The optical micrograph of BP (Fig. 2(a)) revealed its cell structure, clearly showing the cell wall. Fig. 2(b) shows the SEM micrograph of BP biochar, indicating that the size of the cell structure is close to that of BP. This slight change in the cell structure after pyrolysis can be attributed to the biochar production at a slow heating rate (3 °C/min).

**Fig. 3:**
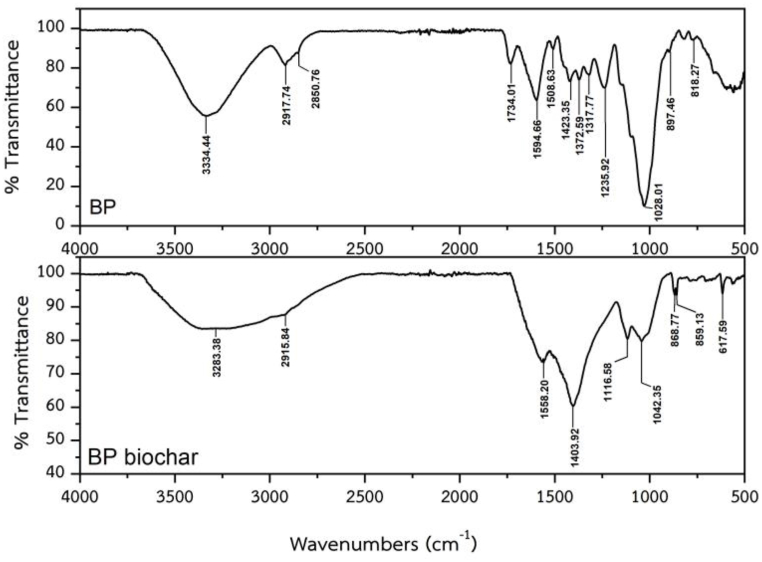
FTIR spectra of the *Biden pilosa* (BP) and *Biden pilosa* biochar (BP biochar).

Fig. 3 shows the FTIR spectra of BP and its biochar. The spectra of BP are similar to those of cotton fibers. For example, the peak at 3334 cm^⁻1^ is a characteristic of the hydroxyl group of cellulose. The peak at 2917 cm^⁻1^ and 2850 cm^⁻1^ is C-H stretching in cellulose. The strong peak at 1028 cm^⁻1^ is the C-O and O-H stretching of polysaccharides in cellulose [33].

For the BP biochar, several adsorption peaks were observed in the FTIR spectrum at 3283, 2915, 1559, 1403, 1116, and 863/839 cm^−1^, which were attributable to the hydroxyl (O–H) stretching, C–H stretching of an alkane, C=C stretching of a cyclic alkane, O–H bending of a carboxylic acid or an alcohol, C–O stretching of an alcohol, and C=C bending of alkene or C–H stretching of an aromatic compound, respectively (Fig. 3).

The FTIR spectrum of BP biochar was similar to those of rice stalk biochar [2,4,20] and *Wodyetia bifurcata* biochar [34].

**Fig. 4:**
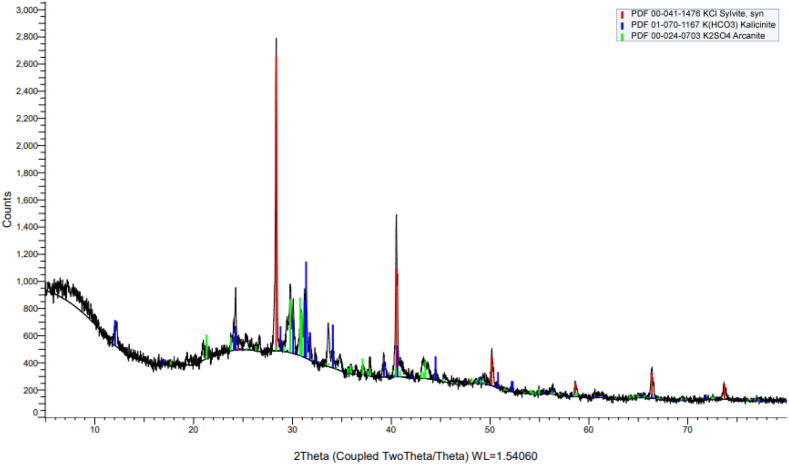
XRD of the *Biden Pilosa* biochar.

The XRD patterns (Fig. 4) of the BP biochar informed the existing inorganic compounds, such as potassium chloride (KCl, 2Ɵ = 28.34°, 40.50°), potassium hydrogen carbonate (K(HCO_3_), 2Ɵ = 31.23°, 34.09°), and potassium sulfate (K_2_SO_4_, 2Ɵ = 30.78° 29.78°). KCl was the phase with small amounts of K(HCO_3_) and K_2_SO_4_. The amounts of inorganic compounds in the BP biochar were very small in the form of ash, which are normally found in charcoal and biochar.

### 3.2 Adsorption response to the initial concentration of the MB solution and the shake period

**Fig. 5:**
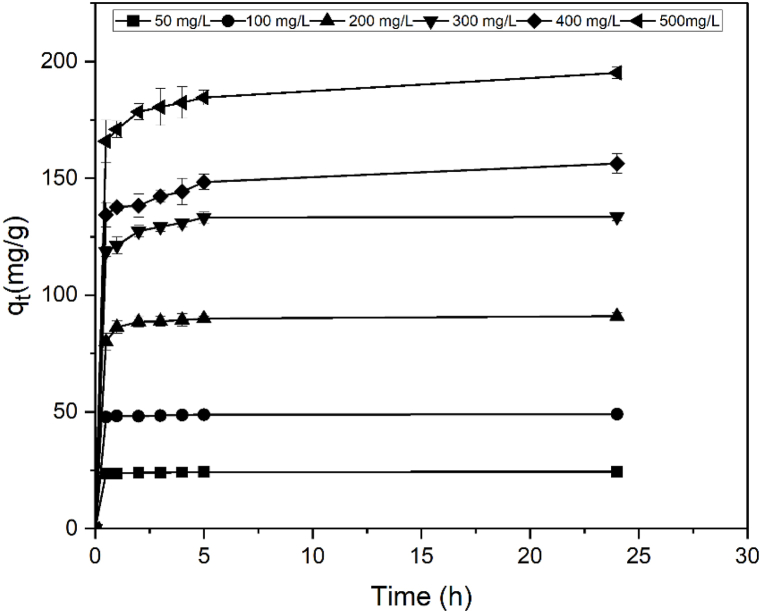
Plot of q_t_ vs. time at various initial MB concentrations for its adsorption on the BP biochar.

The equilibrium period–MB solution concentration relationship was divided into two groups, i.e., low concentration (50–300 mg/l) and high concentration (400–500 mg/l). At a low concentration, it reaches equilibrium after 3 h (Fig. 5); however, those with a high concentration require an equilibrium period of up to 24 h.

The adsorption of MB on the BP biochar increased over time. BP biochar exhibited numerous free adsorption sites at low initial MB concentrations. At a starting concentration of >300 mg/l, the MB molecules’ accumulation could affect the binding rate and orientation of MB on the BP biochar [35]. There was an increase in the adsorption capacity at equilibrium from 25 to 195 mg/g, with the MB concentration increasing from 50 to 500 mg/l, respectively.

This confirms the ability of BP biochar to absorb MB from solutions despite having a small surface area. It is possible that the functional groups on the BP biochar surface (e.g., O–H and C–O) were a contributing factor to the MB's adsorption, which enhanced the overall adsorption process.

**Fig. 6:**
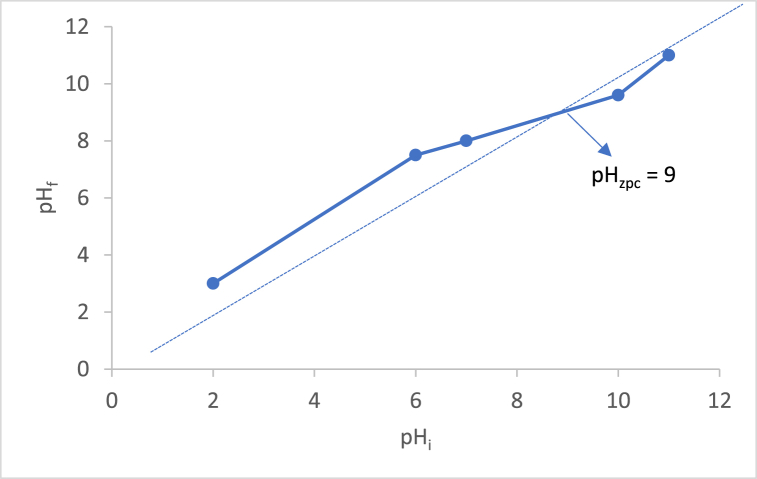
The pHpzc determination curve of BP biochar.

pH affects the adsorption capacity of biochar to dye. The zero point of charge (pHzpc) determines the type of charge on the adsorbents’ surface [8]. Based on Fig. 6, the BP biochar pHzpc was basic. Thus, if the pH of the solution is below 9, the BP biochar surface is positively charged, causing a push against MB, which is a cationic dye. If the pH of the solution is higher than 9, its surface is negatively charged, causing electrostatic interactions and allowing for good adsorption.

**Fig. 7:**
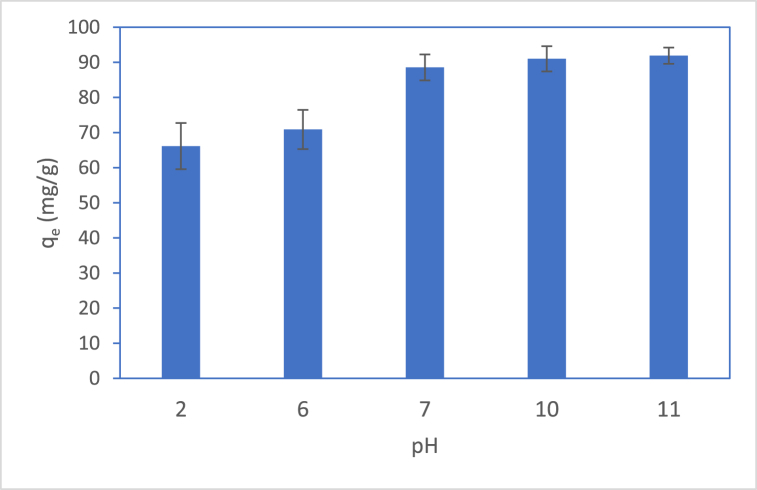
Effect of pH on adsorption capacity of BP biochar.

In this experiment, MB solutions had a pH of 2–11, and the adsorption capacity of BP biochar on MB was 66–92 mg/g (Fig. 7). Based on pHpzc, the adsorption capacity at a basic pH was higher. According to Thai law, the pH range of industrial effluents must be 5.5–9, so BP biochars can be used as an adsorbent in textile industries since at pH 7–9, it had a high adsorption capacity.

There are four possible adsorption mechanisms involved in the adsorption of MB in BP biochars: π-π interaction, hydrogen bonding, electrostatic interaction, and pore filling. For π-π interactions, functional groups (O-H, C = C, and C-O) are regarded as π-electron donors. In terms of hydrogen bonding, the O-H groups on the BP biochar surface play an important role. As pH influences the results, it confirmed the electrostatic interaction between BP biochars and MB. Finally, the pore size of BP biochars was 8 nm, which led to pore filling mechanism.

### 3.3 Isotherm model of the MB adsorption on BP biochar

**Fig. 8:**
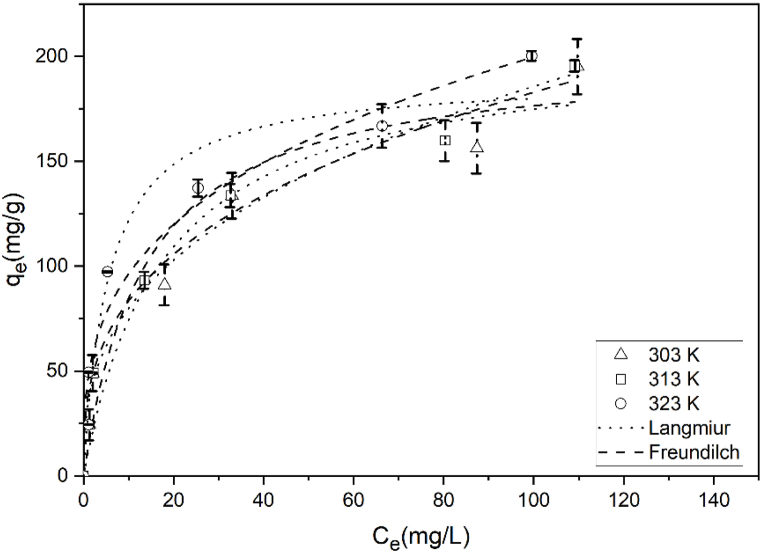
Plot of the adsorption isotherm of MB on the BP biochar at different temperatures.

Fig. 8 shows two theoretical isotherms, illustrating the experimental data points and a comparison. The experimental study results were found to optimally fit the Langmuir model. The MB adsorption capacity of biochar was estimated based on the adsorption isotherms [36].

**Fig. 9:**
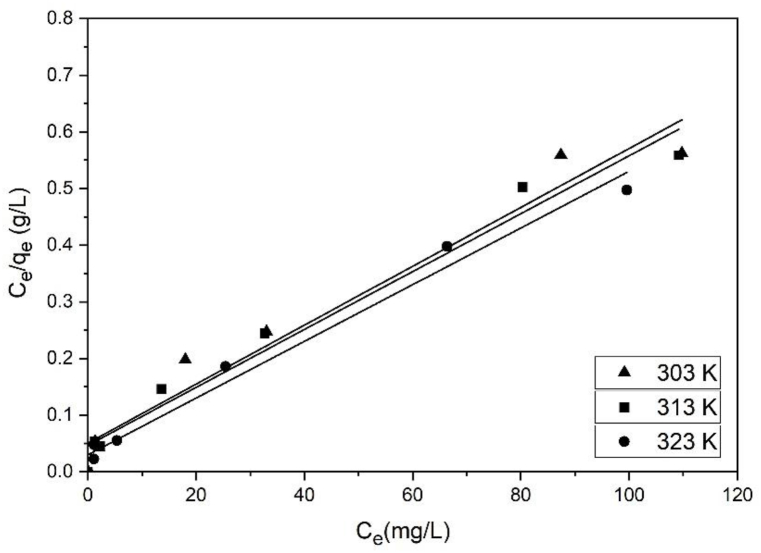
Linear plot of the MB adsorption on the BP biochar following the Langmuir isotherm model.

**Fig. 10:**
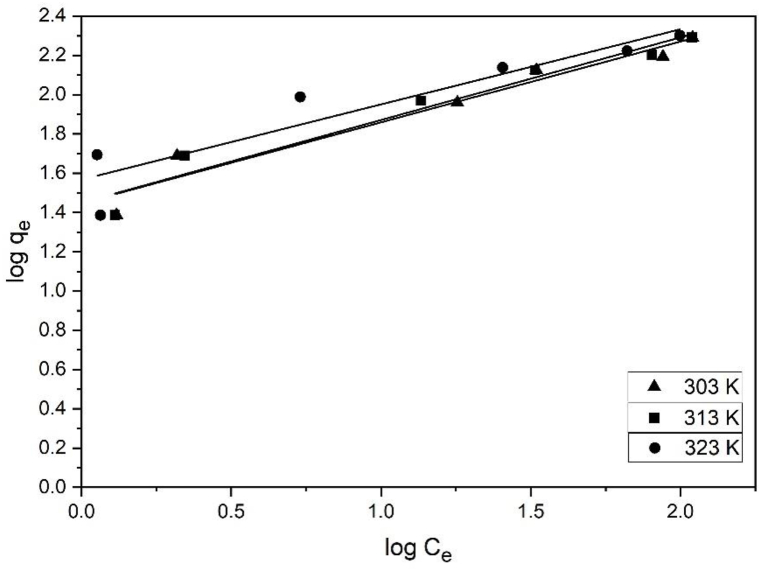
Linear plot of the MB adsorption on the BP biochar following the Freundlich isotherm model.

Table 1 shows the K_L_, q_m_, and R^2^ values for the Langmuir adsorption isotherm model. These values were obtained by plotting C_e_/q_e_ vs. C_e_ (Fig. 9). The BP biochar adsorption capacities for MB were 200, 204, and 204 mg/g at 303, 313, and 323 K, respectively. q_m_ and K_L_ increased with the increase in the solution temperature. This reflects the endothermic activity of the system employing the BP biochar for MB adsorption [20]. The R^2^ value obtained using this model was 0.95–0.97. Therefore, the Langmuir adsorption isotherm suited the MB adsorption on the BP biochar. Table 1 lists the K_F_, n, and R^2^ values for the Freundlich adsorption isotherm model, which were obtained from plotting log q_e_ and log C_e_ (Fig. 10).

**Table 1:** Constants of the Langmuir and Freundlich isotherm models for MB adsorption on the BP biochar at differing temperatures.

T (K)	Langmuir model				Freundlich model			
	q_m_ (mg/g)	K_L_ (mg/L)	R^2^	RMSE	K_F_ [(mg/g) (mg/L)^1/n^]	n	R^2^	RMSE
303	200	0.074	0.95	10.37	28	2.42	0.97	10.13
313	204	0.079	0.97	9.92	28.2	2.37	0.98	6.14
323	204	0.126	0.96	9.89	37	2.61	0.95	7.18

A good adsorption typically has a Freundlich constant n between 1 and 10, whereas 1/n = 1 reflects a linear adsorption, resulting in identical adsorption energies at all sites. Larger n values (lower values of 1/n) imply a more powerful adsorbent–adsorbate interaction. Typically, the occurrence of linear adsorption manifests at particularly low concentrations of solute and adsorbent loadings [37]. Here, the n value of the Freundlich model, ranging from 1 to 10, indicated the successful MB adsorption on the BP biochar.

The R^2^ value obtained using the Freundlich isotherm model was 0.95–0.98. The Freundlich adsorption isotherm was also appropriate for MB adsorption on BP biochar. Nevertheless, the Langmuir isotherm's somewhat different experimental and calculated q_e_ data showed a greater RMSE (6.14–10.13) than the Freundlich isotherm's RMSE (9.89–10.37).

The comparative results of the Langmuir-based maximum MB adsorption capacity of a range of adsorbents are listed in Table 2. The BP biochar developed in this work exhibited a higher adsorption capacity than rice stalk, sugarcane, and switchgrass biochars within the same range of pyrolysis temperature (<600 °C). The adsorption capacity of BP biochar is close to that of the biochar based on date palm fronds. However, the BP biochar was prepared at a lower pyrolysis temperature than that used to prepare date palm biochar. This confirms that the type of biomass used to produce biochar affects the physical properties of the produced biochar, especially its adsorption capacity.

**Table 2:** Comparison of the maximum Langmuir-based MB dye adsorption capacities of different adsorbents.

Adsorbent	Pyrolysis temperature (°C)	Adsorption capacity (mg/g)	References
*Biden pilosa* biochar	550	200	This work
Rice stalk biochar	500	90.91	[2]
Sugarcane bagasse biochar	500	113	[16]
Switchgrass biochar	600	37.6	[38]
Date palm fronds biochar	700	205	[14]
*Wodyetia bifurcata*-based activated carbon	700	149.30	[34]
Longan seed-based activated carbon	850	1000	[12]

These findings are similar to those obtained by Yu K.L [39] using *Chlorella* sp. microalgae biochar, which exhibited an MB adsorption capacity of 113 mg/g and a 2.65 m^2^/g surface area. To increase the carbon or biochar's surface area and enhance their adsorption capacities, some studies focus on activation processes. However, the increase in the surface area does not always result in an increase in the adsorption capacity. For example, FeCl_3_-activated coffee husk exhibited a 965 m^2^/g surface area and a 71 mg/g MB adsorption capacity [40].

### 3.4 Kinetic model of the MB adsorption on BP biochar

**Fig. 11:**
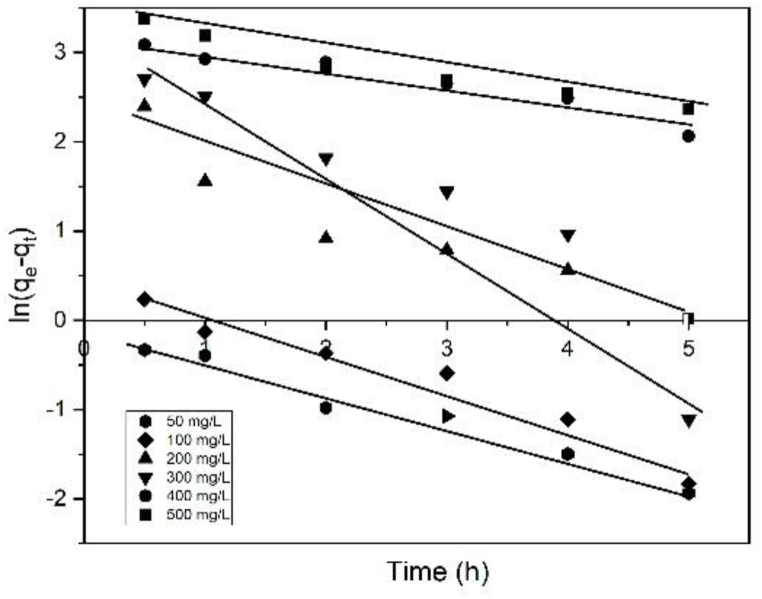
Linear plot of the MB adsorption on the BP biochar at 303 K following the pseudo-first-order kinetics model.

**Fig. 12:**
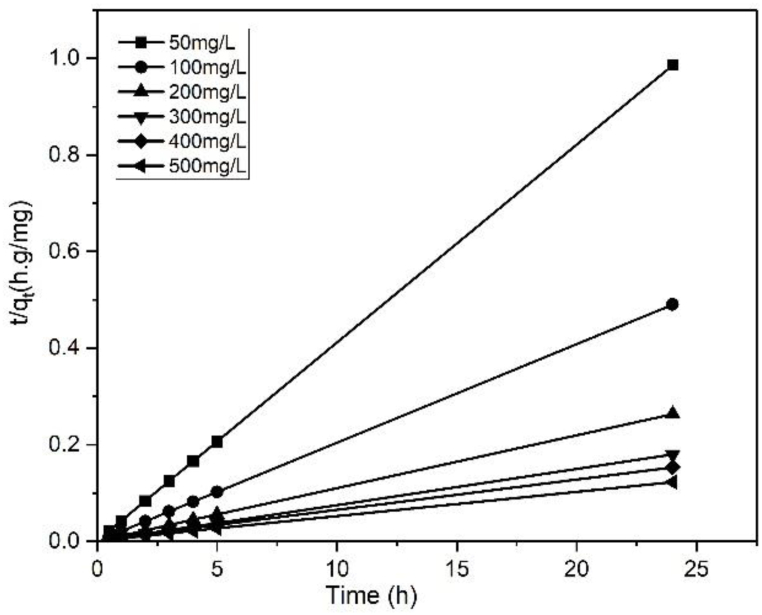
Linear plot of the MB adsorption on the BP biochar at 303 K following the pseudo-second-order kinetics model.

Fig. 11 shows the ln (q_e_–q_t_) vs. time curve at various MB concentrations. This figure was used to analyze the adsorption kinetics and determine the values for k_1_ and q_e,cal_, while Table 3 lists the k_1_, q_e,cal_, and R^2^ values. The values for q_e,exp_ and q_e,cal_ were different despite R^2^ being higher than 0.8. Thus, the MB adsorption on the BP biochar was not a first order kinetic process. Fig. 12 shows the linear plot between t/q_t_ and time for the pseudo-second-order kinetic model. The k_2_, q_e,cal_, and R^2^ values of the model are listed in Table 3.

**Table 3:** Adsorption capacity and pseudo-first/second order rate constants for various initial MB concentrations at 303 K.

C_o_ (mg/L)	q_e, exp_ (mg/g)	First order kinetic model				Second-order kinetic model			
		k_1_ (h^−1^)	q_e, cal_ (mg/g)	R^2^	RMSE	k_2_ (g/mg h)	q_e,cal_ (mg/g)	R^2^	RMSE
50	24.35	0.3526	0.89	0.97	10.49	1.201	24.39	1	0.01
100	48.96	0.4148	1.50	0.95	21.22	0.833	49.01	1	0.02
200	91.00	0.4491	9.00	0.88	36.67	0.151	90.90	1	0.04
300	133.50	0.7474	27.60	0.88	47.56	0.093	133.33	1	0.07
400	156.30	0.2059	24.91	0.94	58.76	0.024	158.73	0.99	1.08
500	195.13	0.2162	29.63	0.95	74.01	0.023	196.07	0.99	0.42

The t/q vs. t linear plots (Fig. 12) demonstrate acceptable calculated–experimental q_e_ value agreement (Table 3). The equilibrium adsorption capacity, q_e,cal_, derived utilizing the second-order kinetic model is compatible with the experimental data, q_e,exp_, and its correlation coefficients are higher than 0.99 with a small RMSE. Therefore, it is suggested that the adsorption of MB is governed by the pseudo-second-order kinetic model. A better model fitting results in a higher R^2^ value and a lower RMSE value [8]. Table 3 shows that the MB adsorption on BP biochar can be adequately represented by the second-order kinetic model. This work is comparable to the MB adsorption on rice straw and switchgrass biochar [2,38].

### 3.5 Adsorption thermodynamics

**Fig. 13:**
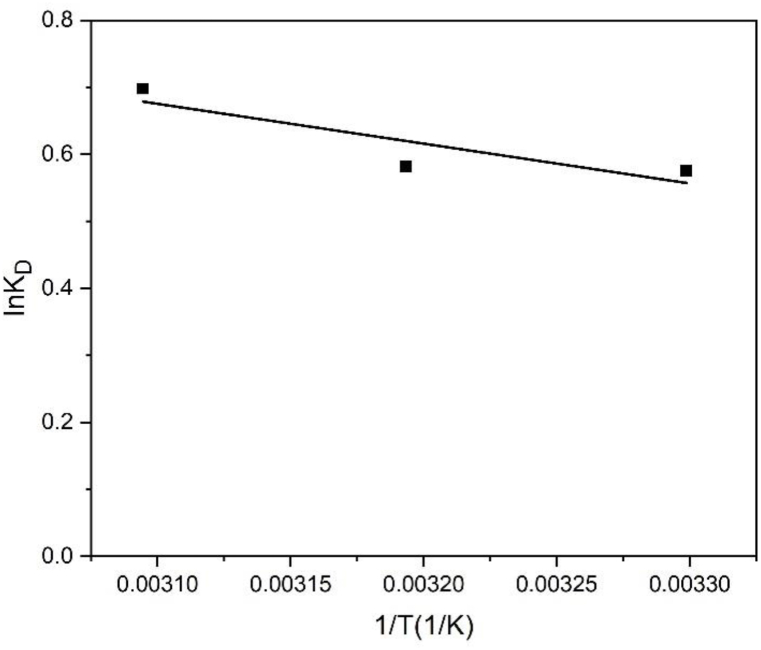
Plot of the relationship between lnK_D_ and 1/T for the adsorption process's enthalpy change.

The MB solution at an initial concentration of 500 mg/l was used to study adsorption thermodynamics. The relation between lnK_D_ and 1/T for MB adsorption on BP biochar is shown in Fig. 13 and linear fitting (R^2^ = 0.80) of lnK_D_ and 1/T was obtained. The thermodynamic parameters of the adsorption process are listed in Table 4. H^0^ was approximately 4.94 kJ/mol. This positive value denotes an endothermic reaction, and its magnitude reflects a physisorption when H^0^ is less than 50 kJ/mol [41]). Based on previous studies on biochar, the positive value of S^0^ obtained here (S^0^ = 0.02 kJ/mol K) reflects the increased variability of the interface between the solid (adsorption media) and solution throughout the adsorption [8,34]. Based on the negative values of G^0^ obtained at 303, 313, and 323 K, the process became more feasible, spontaneous, and favorable with the increase in temperature [34,42].

**Table 4:** Thermodynamic parameters for the adsorption of MB on BP biochar.

ΔH^0^ (kJ/mol)	ΔS^0^ (kJ/mol K)	ΔG^0^ (kJ/mol)		
		303 K	313 K	323 K
4.94	0.02	−1.41	−1.61	−1.82

### 3.6 Cost estimation

BP biochars showed some advantages, as follows;

1. The drum kiln-HDP costs 1000 US$.
2. Nitrogen flow is not needed during production because it is a charcoal kiln.
3. The kiln is movable, so it can be used in the field without transferring feedstocks.
4. There was no sample pretreatment before loading. BP with the same size was used, which was cut from the field.
5. BP biochar was easy to grind, so energy for BP biochar size reduction was conserved.

Table 5 and Table 6 show the estimated direction cost for the biochar production and the estimated income for BP biochar, respectively. There was no raw material expense because BP was an agricultural waste that was cut by a worker. For BP biochar production, one worker was able to operate two kilns at the same time. Therefore, the labor cost was from one worker in a day. The BP biochar from one kiln was 0.8 kg. This means the BP biochar from two kilns was 1.6 kg. If the price of BP biochar was 9 USD/kg, the income from BP biochar was 14.4 USD, or the profit was 4.4 USD for production. Then BP biochar is possible for commercialization. According to online marketing information, the sale price of activated carbon powder was about 25–96 USD/kg.

**Table 5:** Estimate of direct production cost for the BP biochar production.

Direct cost item	Amount	Amount of money (USD)	Remark
Raw material			
*Biden pilosa*	44 kg	–	For two kilns
Direct labor cost	1 worker	10	Labor wage 10 USD/day
Production expense			
Indirect labor cost	–	–	
Public utility cost	–	–	
Total production expense	–	–	
Total production cost		10

**Table 6:** Estimate income statement for BP biochar.

Items	Amount	Amount of money (USD)	Remark
BP biochar	1.6 kg	14.4	BP biochar price: 9 USD/kg

This study offers basic information for producing effective biochar for MB adsorption using a drum kiln with a heat-transferring duct, in which the pyrolysis temperature can be controlled. The BP biochar was not activated, making this an energy and chemical-saving method. Therefore, it is possible to produce BP biochar in local areas for *in situ* water treatment, which is a huge advantage because the cost of transportation is a significant element in the economics of producing biochar [43].

## 4 Conclusion

Non-activated BP biochars were successfully produced from fresh BP on a pilot scale in an agricultural area. The carbonization step took 5 h to reach the maximum pyrolysis temperature at 550 °C. High-quality biochars can be produced using the proposed method, which involves only modest devices without the need for sophisticated technologies. Various concentrations of MB were removed from aqueous solutions using non-activated BP biochar as an adsorbent. A monolayer Langmuir isotherm was used to describe the adsorption behavior. A pseudo-second-order kinetic model was used to describe the kinetic data. The value obtained from the Langmuir model was comparable to the maximum adsorption capacity, q_m_ (200 mg/g), at 303 K.

## Author contribution statement

Supin Sangsuk: Conceived and designed the experiments; Performed the experiments; Analyzed and interpreted the data; Contributed reagents, materials, analysis tools or data; Wrote the paper.

Pinanong Napanya, Siwabhorn Tasen, Phannida Baiya, Chatchai Buathong, Khemissara Keeratisoontornwat: Performed the experiments.

Sirisak Suebsiri: Contributed reagents, materials, analysis tools or data.

## Data availability statement

Data will be made available on request.

## Declaration of interest's statement

The authors declare no competing interests.

## Declaration of competing interest

The authors declare that they have no known competing financial interests or personal relationships that could have appeared to influence the work reported in this paper.

## References

[1] Mtenga D.V., Ripanda A.S. (2022). A review on the potential of underutilized Blackjack (Biden Pilosa) naturally occurring in sub-Saharan Africa. Heliyon.

[2] Abd-Elhamid A.I., Emran M., El-Sadek M.H., El-Shanshory A.A., Soliman H.M.A., Akl M.A., Rashad M. (2020). Enhanced removal of cationic dye by eco-friendly activated biochar derived from rice straw. Appl. Water Sci..

[3] Bhatnagar A., Hogland W., Marques M., Sillanpää M. (2013). An overview of the modification methods of activated carbon for its water treatment applications. Chem. Eng. J..

[4] Li X., Li Y. (2019). Adsorptive removal of dyes from aqueous solution by KMnO_4_-modified rice husk and rice straw. J. Chem..

[5] Thompson K.A., Shimabuku K.K., Kearns J.P., Knappe D.R.U., Summers R.S., Cook S.M. (2016). Environmental comparison of biochar and activated carbon for tertiary wastewater treatment. Environ. Sci. Technol..

[6] Wang X., Zhou W., Liang G., Song D., Zhang X. (2015). Characteristics of maize biochar with different pyrolysis temperatures and its effects on organic carbon, nitrogen and enzymatic activities after addition to fluvo-aquic soil. Sci. Total Environ..

[7] Janu R., Mrlik V., Ribitsch D., Hofman J., Sedláček P., Bielská L., Soja G. (2021). Biochar surface functional groups as affected by biomass feedstock, biochar composition and pyrolysis temperature. Carbon Resour. Conv..

[8] Ahmed M.J., Okoye P.U., Hummadi E.H., Hameed B.H. (2019). High-performance porous biochar from the pyrolysis of natural and renewable seaweed (*Gelidiella acerosa*) and its application for the adsorption of methylene blue. Bioresour. Technol..

[9] Parthasarathy P., Sajjad S., Saleem J., Alherbawi M., Mckay G. (2022). A review of the removal of dyestuffs from effluents onto biochar. Separations.

[10] Chung K.T. (2016). Azo dyes and human health: a review. J. Environ. Sci. Health C Environ. Carcinog. Ecotoxicol. Rev..

[11] Lu G., Nagbanshi M., Goldau N., Mendes Jorge M., Meissner P., Jahn A., Mockenhaupt F.P., Müller O. (2018). Efficacy and safety of methylene blue in the treatment of malaria: a systematic review. BMC Med..

[12] Lawtae P., Tangsathitkulchai C. (2021). The use of high surface area mesoporous-activated carbon from longan seed biomass for increasing capacity and kinetics of methylene blue adsorption from aqueous solution. Molecules.

[13] Patawat C., Silakate K., Chuan-Udom S., Supanchaiyamat N., Hunt A.J., Ngernyen Y. (2020). Preparation of activated carbon from *Dipterocarpus alatus* fruit and its application for methylene blue adsorption. RSC Adv..

[14] Zubair M., Mu’azu N.D., Jarrah N., Blaisi N.I., Aziz H.A., Al-Harthi M.A. (2020). Adsorption behavior and mechanism of methylene blue, crystal violet, eriochrome black T, and methyl orange dyes onto biochar-derived date palm fronds waste produced at different pyrolysis conditions. Water Air Soil Pollut..

[15] Yin Z., Liu N., Bian S., Li J., Xu S., Zhang Y. (2019). Enhancing the adsorption capability of areca leaf biochar for methylene blue by K2FeO4-catalyzed oxidative pyrolysis at low temperature. RSC Adv..

[16] Biswas S., Mohapatra S.S., Kumari U., Meikap B.C., Sen T.K. (2020). Batch and continuous closed circuit semi-fluidized bed operation: removal of MB dye using sugarcane bagasse biochar and alginate composite adsorbents. J. Environ. Chem. Eng..

[17] Suhaimi N., Kooh M.R.R., Lim C.M., Chou Chao C.T., Chou Chau Y.F., Mahadi A.H., Chiang H.P., Haji Hassan N.H., Thotagamuge R. (2022). The use of gigantochloa bamboo-derived biochar for the removal of methylene blue from aqueous solution. Adsorpt. Sci. Technol..

[18] Li L., Zou D., Xiao Z., Zeng X., Zhang L., Jiang L., Wang A., Ge D., Zhang G., Liu F. (2019). Biochar as a sorbent for emerging contaminants enables improvements in waste management and sustainable resource use. J. Clean. Prod..

[19] Ahmad M., Rajapaksha A.U., Lim J.E., Zhang M., Bolan N., Mohan D., Vithanage M., Lee S.S., Ok Y.S. (2014). Biochar as a sorbent for contaminant management in soil and water: a review. Chemosphere.

[20] Sackey E.A., Song Y., Yu Y., Zhuang H. (2021). Biochars derived from bamboo and rice straw for sorption of basic red dyes. PLoS One.

[21] Sangsuk S., Buathong C., Suebsiri S. (2020). High-energy conversion efficiency of drum kiln with heat distribution pipe for charcoal and biochar production. Energy Sustain. Dev..

[22] Feldsine P., Abeyta C., Andrews W.H. (2002). AOAC International methods committee guidelines for validation of qualitative and quantitative food microbiological official methods of analysis. J. AOAC Int..

[23] Langmuir I. (1916). The constitute and fundamental properties of solids and liquids. Part I. Solids. J. Am. Chem. Soc..

[24] Freundlich H. (1906). Over the adsoption in solution. J. Phys. Chem..

[25] Lagergren S. (1898). Zur theorie der sogenannten adsorption geloster stoffeitle, Kungliga Svenska Vetenskapsakademiens. Handlingar.

[26] Ho Y.S., McKay G. (1998). Sorption of basic dye from aqueous solution by pomelo. Chem. Eng. J..

[27] Lakshmi U.R., Srivastava V.C., Mall I.D., Lataye D.H. (2009). Rice husk ash as an effective adsorbent: evaluation of adsorptive characteristics for Indigo Carmine dye. J. Environ. Manag..

[28] Sun H., Qin Y., Liu X., Li H. (2018). Natural mesoporous activated carbon from toxic plant stellera chamaejasme roots by chemical methods. J. Bioresour. Bioprod..

[29] Jawad A.H., Hum N.N.M.F., Farhan A.M., Mastuli M.S. (2020). Biosorption of methylene blue dye by rice (*Oryza sativa* L.) straw: adsorption and mechanism study. Desalination Water Treat..

[30] Spagnoli A.A., Giannakoudakis D.A., Bashkova S. (2017). Adsorption of methylene blue on cashew nut shell based carbons activated with zinc chloride: the role of surface and structural parameters. J. Mol. Liq..

[31] Elnour A.Y., Alghyamah A.A., Shaikh H.M., Poulose A.M., Al-Zahrani S.M., Anis A., Al-Wabel M.I. (2019). Effect of pyrolysis temperature on biochar microstructural evolution, physicochemical characteristics, and its influence on biochar/polypropylene composites. Appl. Sci..

[32] Xiao H., Lin Q., Li G., Zhao X., Li J., Li E. (2022). Comparison of biochar properties from 5 kinds of halophyte produced by slow pyrolysis at 500°C. Biochar.

[33] Portella E.H., Romanzini D., Angrizani C.C., Amico S.C., Zattera A.J. (2016). Influence of stacking sequence on the mechanical and dynamic mechanical properties of cotton/glass fiber reinforced polyester composites. Mater. Res..

[34] dos Santos K.J.L., de Souza dos Santos G.E., de Sá Í.M.G.L., Ide A.H., da Silva Duarte J.L., de Carvalho S.H.V., Soletti J.I., Meili L. (2019). Wodyetia bifurcata biochar for methylene blue removal from aqueous matrix. Bioresour. Technol..

[35] Güzel F., Sayğılı H., Akkaya Sayğılı G., Koyuncu F., Yılmaz C. (2017). Optimal oxidation with nitric acid of biochar derived from pyrolysis of weeds and its application in removal of hazardous dye methylene blue from aqueous solution. J. Clean. Prod..

[36] Giusto L.A.R., Pissetti F.L., Castro T.S., Magalhães F. (2017). Preparation of activated carbon from sugarcane bagasse soot and methylene blue adsorption. Water Air Soil Pollut..

[37] Anah L., Astrini N. (2018). Isotherm adsorption studies of Ni(II) ion removal from aqueous solutions by modified carboxymethyl cellulose hydrogel. IOP Conf. Ser. Earth Environ. Sci..

[38] Park J.H., Wang J.J., Meng Y., Wei Z., DeLaune R.D., Seo D.C. (2019). Adsorption/desorption behavior of cationic and anionic dyes by biochars prepared at normal and high pyrolysis temperatures. Colloids Surf. A Physicochem. Eng. Asp..

[39] Yu K.L., Lee X.J., Ong H.C., Chen W.H., Chang J.S., Lin C.S., Show P.L., Ling T.C. (2021). Adsorptive removal of cationic methylene blue and anionic Congo red dyes using wet-torrefied microalgal biochar: equilibrium, kinetic and mechanism modeling. Environ. Pollut..

[40] Oliveira L.C.A., Pereira E., Guimaraes I.R., Vallone A., Pereira M., Mesquita J.P., Sapag K. (2009). Preparation of activated carbons from coffee husks utilizing FeCl_3_ and ZnCl_2_ as activating agents. J. Hazard Mater..

[41] Worch E. (2021). Adsorption Technology in Water Treatment.

[42] Fan S., Wang Y., Wang Z., Tang J., Tang J., Li X. (2017). Removal of methylene blue from aqueous solution by sewage sludge-derived biochar: adsorption kinetics, equilibrium, thermodynamics and mechanism. J. Environ. Chem. Eng..

[43] Oni B.A., Oziegbe O., Olawole O.O. (2019). Significance of biochar application to the environment and economy. Ann. Agric. Sci..

